# Direct observation of deterministic domain wall trajectory in magnetic network structures

**DOI:** 10.1038/srep19027

**Published:** 2016-01-12

**Authors:** P. Sethi, C. Murapaka, S. Goolaup, Y. J. Chen, S. H. Leong, W. S. Lew

**Affiliations:** 1 School of Physical & Mathematical Sciences, Nanyang Technological University, 21 Nanyang Link, Singapore 637371; 2 Data Storage Institute, (A*STAR) Agency for Science, Technology and Research, DSI Building, 5 Engineering Drive 1, Singapore 117608.

## Abstract

Controlling the domain wall (DW) trajectory in magnetic network structures is crucial for spin-based device related applications. The understanding of DW dynamics in network structures is also important for study of fundamental properties like observation of magnetic monopoles at room temperature in artificial spin ice lattice. The trajectory of DW in magnetic network structures has been shown to be chirality dependent. However, the DW chirality periodically oscillates as it propagates a distance longer than its fidelity length due to Walker breakdown phenomenon. This leads to a stochastic behavior in the DW propagation through the network structure. In this study, we show that the DW trajectory can be deterministically controlled in the magnetic network structures irrespective of its chirality by introducing a potential barrier. The DW propagation in the network structure is governed by the geometrically induced potential barrier and pinning strength against the propagation. This technique can be extended for controlling the trajectory of magnetic charge carriers in an artificial spin ice lattice.

Recent developments in domain wall (DW) dynamics in magnetic network structures have enabled the study of magnetic charge carrier hopping in artificial spin ice lattice^1^,^2^,^3^,^4^,^5^. Observation of magnetic monopoles and other interesting thermodynamic properties^1^,^2^,^3^,^4^,^5^,^6^,^7^,^8^ has motivated recent research interest in artificial spin ice network structures. The trajectory of DWs in these network structures assists in understanding the underlying magnetization dynamics. The creation and absorption of DWs at the vertex of the network structure governs the net magnetic charge and its trajectory in the artificial spin ice lattice^9^. Recently, Zeissler *et al.* reported a non-random walk of charge carriers in artificial spin ice lattice, which was attributed to the DW following a chirality dependent path through the network^10^. Pushp *et al.* reported a selective switching of vortex DW (VDW) in a Y-shaped branch structure. The selective switching was ascribed to the arrangement of topological edge charges of DW at the bifurcation of the network structure^11^. We previously reported selective trajectory of a transverse DW (TDW) in Y-shaped branch structure via a complex interaction of edge defects with the vertex at the bifurcation^12^. The above observations have direct application in the formation of 1D Dirac strings in an artificial spin ice lattice^2^,^11^. The deterministic trajectory of magnetic charge carriers in spin ice structure is dependent on the robustness of the DW chirality. At low magnetic field strengths, the DW preserves its chirality and spin structure^13^, and the wall velocity increases linearly with the magnetic field^14^. However, above a critical field (Walker field) H_w_, the average DW velocity reduces drastically^14^,^15^ due to periodic transformation of the DW spin structure between Néel and Bloch configurations. This phenomenon, which is termed as Walker breakdown, leads to complex transformations of DW structure during its propagation^13^. Burn *et al.* reported that the path taken by a DW in a dual-branch network structure alternated between the upper and lower branch at relatively high magnetic field strengths^16^. In this work, a geometrical asymmetry has been introduced in magnetic network structures to control the trajectory of the DW without the restriction of the chirality. It is shown that asymmetry provides a potential barrier to the DW and constrains it to move in a particular branch, making the output immune to the effect of Walker breakdown. This technique allows the control of the DW trajectory in an artificial spin ice lattice, irrespective of its chirality.

## Results

**Figure 1:**
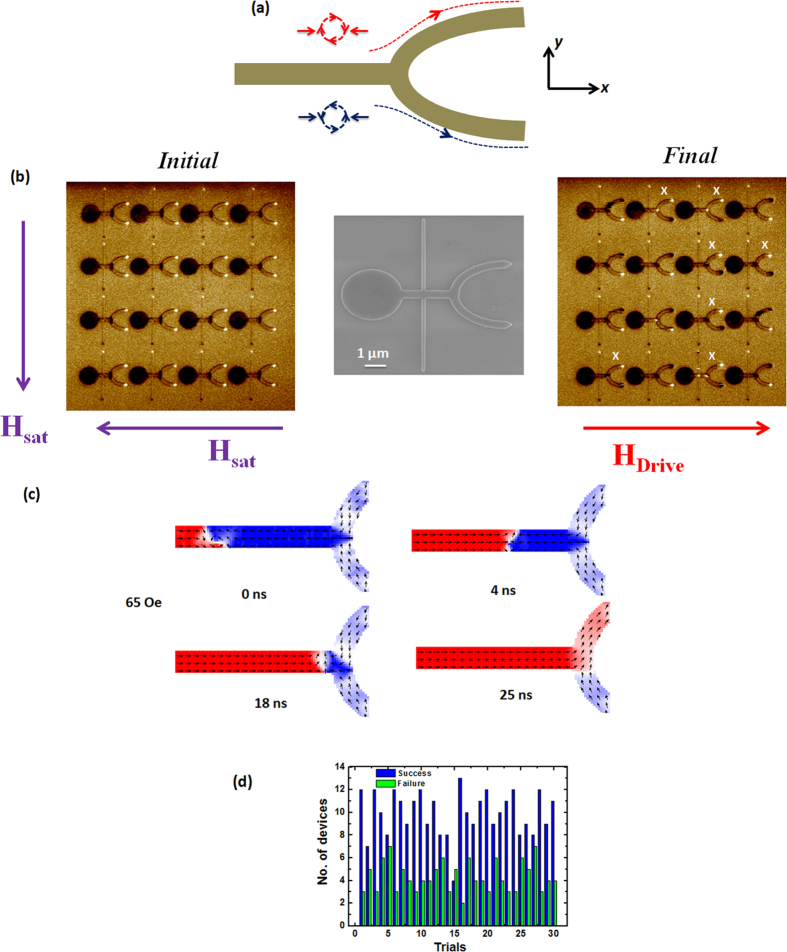
Trajectory of vortex domain walls in branch structures. (**a**) Schematic of ‘U’ shaped branch structure showing the trajectory of clockwise (CW) vortex domain wall (VDW) along the upper branch and anticlockwise (ACW) VDW along the lower branch. (**b**) Scanning electron microscopy (SEM) image of the ‘U-shaped’ branch structure shown in the middle used for studying field induced DW motion. Show on the left is the magnetic force microscopy (MFM) image of the initial magnetization configuration of an array of branch structures when the transverse nanowire and output branch are saturated along the −*y* and −*x* direction, respectively. Shown on the right of SEM is the final magnetization configuration of the array structure when a head-to-head clockwise (HH-CW) VDW is injected and driven. The results indicate that the DW selects the output branch randomly. (**c**) Micromagnetic simulations depicting the VDW motion along the +*x* direction in the branch structure at magnetic field strength of 65 Oe. The DW moves in opposite branches for the two field strengths. (**d**) Relative distribution of successful and failed trials when a DW is propagated in a symmetric structure. The success in a trial implies that the DW follows a selective trajectory governed by its initial chirality.

Figure 1(a) shows schematic of a branch structure chosen to verify the chirality dependent DW trajectory. For a head-to-head (HH) DW, in which the spins point towards each other, a clockwise (CW) chirality VDW is expected to propagate along the upper branch whereas an anticlockwise chirality (ACW) VDW moves along the lower branch^11^,^17^. Figure 1(b) shows the scanning electron microscopy (SEM) image of a test branch structure. The branch structure was patterned on a thin film of Ta(3 nm)/Ni_81_Fe_19_(10 nm)/Ta(3 nm). The structure was fabricated using electron beam lithography and Ar ion milling techniques. The branch structure comprises of a circular pad of diameter 2 μm, a longitudinal nanowire of width 300 nm, and a ‘U’ shaped branch of width 300 nm. The circular pad is used for nucleating and injecting a DW into the longitudinal nanowire. A 6-μm-long and 100-nm-wide transverse nanowire is positioned at a distance of 1 μm from the output branch to assign chirality to the injected DW^12^,^18^. When the magnetization of the transverse nanowire is aligned along the +*y* direction, the injected DW attains an ACW chirality, conversely, a CW chirality DW is injected if the magnetization is aligned along the −*y* direction. Further details on the setting of DW chirality by a transverse nanowire are shown in supplementary Fig. S1. Magnetic force microscopy (MFM) imaging was carried out on an array of structures to determine the trajectory of field-driven DW into the branch. Figure 1(b) shows MFM images of the initial and final magnetization states of the structures. In the initial state, the magnetization orientation of the transverse nanowire and the branch structure were set along the −*y* and −*x* directions, respectively. This is confirmed by the dark and bright contrasts at the edges of the transverse nanowire and the output branch in the MFM image, that in our convention, the magnetization is always aligned from bright to dark contrast. As the external magnetic field is gradually increased along the +*x* direction, a HH DW with a CW chirality is injected and driven in the longitudinal nanowire. According to the chirality dependent motion, the DW is expected to move along the upper branch^17^ and switch its magnetization which is reflected by the change in magnetic contrast from bright to dark. However, MFM image of the final state of the structures shows that the DW trajectory into the output branch was random. In some of the structures the DW propagated along the lower branch (shown by ‘X’ marks). This observation points to the possibility that the DW chirality is not always preserved during DW motion along the nanowire. We performed 30 MFM scans to get an estimate of the number of successful and failed trials. We observed that in 70% of the trials the DW followed a deterministic trajectory governed by its initial chirality. Figure 1(d) shows the relative distribution of successful and failed trials when a DW propagates in the symmetric structure. The success is achieved in a trial when the DW follows a selective trajectory governed by its initial chirality. We performed t-test to ascertain the 95% confidence level. The range of successful trials was found to be 65%–75%. Thus the trajectory is not completely random however the success rate of selective trajectory based on initial chirality is not very high. To investigate the effect of Walker breakdown on the DW trajectory in a branch structure, micromagnetic simulations^19^ were carried out. Figure 1(c) depicts the simulated magnetization configurations of the DW as a function of the simulation time when driven by applied field strength of 65 Oe. This field value is same as the one used to inject the DW experimentally which is shown later. The initial chirality of the DW is ACW. As the DW propagates, it breaks and transforms into an anti-vortex configuration which eventually transforms to a transverse DW before entering the bifurcation. The DW propagates along the upper branch contrary to what is expected^11^. These results indicate that the DW transformations due to Walker breakdown leads to the DW propagating into an arbitrary branch.

**Figure 2:**
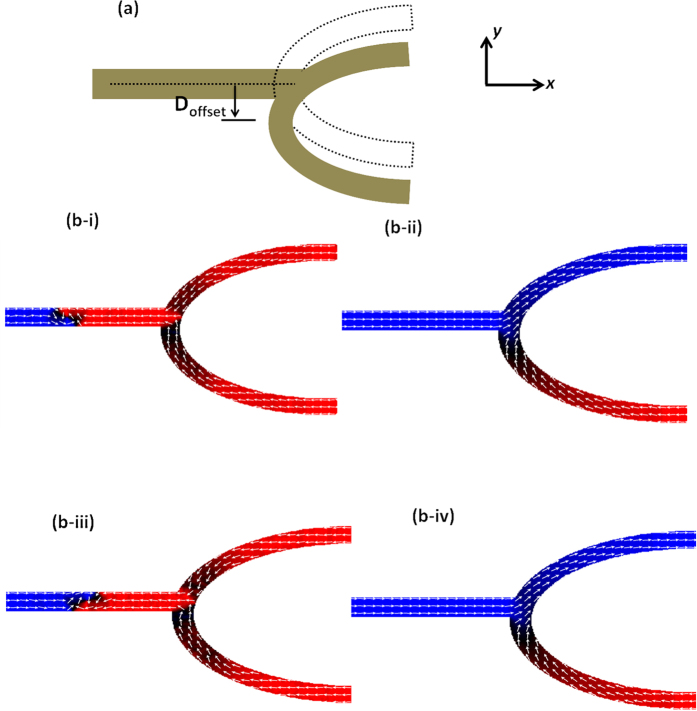
Trajectory of a domain wall in asymmetric branch structures. (**a**) Schematic of ‘pull-down’ (PD) structure, where output branch is displaced in the −*y* direction and the offset is labeled as D_offset_. (**b-i**) Simulated initial magnetization configuration of the PD structure when HH-ACW is injected in the nanowire. (**b-ii**) Simulated final magnetization configuration of the PD structure depicting DW moving to the upper branch. (**b-iii**) Simulated initial magnetization configuration of the PD structure when HH-CW is injected in the nanowire. (**b-iv**) Simulated final magnetization configuration of the PD structure depicting DW moving to the upper branch.

The issue of stochastic behavior of the DW trajectory into the branch structure was overcome by introducing an asymmetry in the structure. Figure 2(a) shows schematic of the structure where the output branch has been displaced in the −*y* direction, this configuration is labeled as ‘pull-down’ (PD). D_offset_ represents the distance by which the branch has been displaced from the center of the longitudinal nanowire. The simulation results for D_offset_ = 200 nm when CW & ACW DWs are driven in this structure are shown in Fig. 2b (i–iv). Figure 2b (i) depicts the initial magnetization state of the structure with an ACW VDW before driving. Figure 2b (ii) represents the final magnetization state of the structure, showing that the magnetization of the upper branch switched from −*x* to +*x* direction. This implies that the ACW VDW propagates along the upper branch. Figure 2b (iii) depicts the initial magnetization state of the structure with a CW VDW before driving. The CW VDW also moves in the upper branch as shown in Fig. 2b (iv). The results reveal that irrespective of the chirality, the DW propagates along the upper branch. Alternatively, the branch can be displaced in the +*y* direction, this structure is labeled as ‘pull up’ (PU) structure. With the displacement along the +*y* direction, the DW moves along the lower branch irrespective of its chirality. The simulation results for PU structure are shown in supplementary Fig. S2.

**Figure 3:**
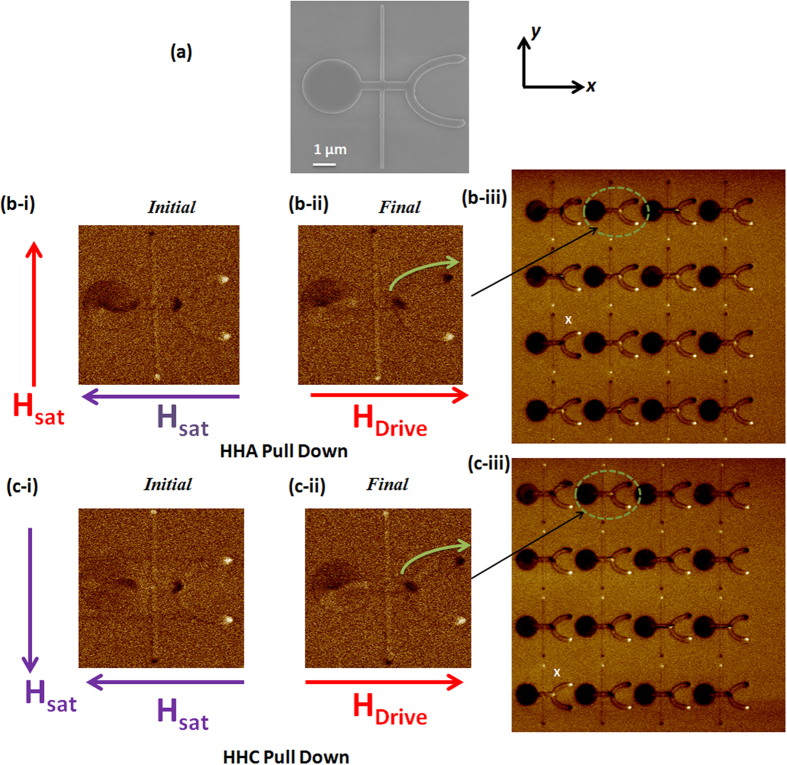
Direct observation of vortex domain wall trajectory in the PD structure. (**a**) SEM image shows the PD structure, the output branch is displaced in the −*y* direction by 200 nm. (**b–i**) MFM image of initial magnetization configuration when transverse nanowire is saturated in the +*y* direction and the output branch is saturated in the −*x* direction. (**b-ii**) MFM image of final magnetization configuration when HH-ACW is driven through the branch structure, (**b-iii**) MFM image of final magnetization configuration image of an array of PD structures, here except one device all other devices DW moves along the upper branch. (**c-i**) MFM image of initial magnetization configuration when transverse nanowire is saturated in the −*y* direction and the output branch is saturated in the −*x* direction. (**c-ii**) MFM image of final magnetization configuration when HH-CW is driven through the branch structure. (**c-iii**) MFM image of an array of PD structure, similar to the previous case the DW moves along the upper branch in majority of the structures.

Experimental study was carried out on DW motion in the asymmetric branch structures using MFM imaging technique to validate the simulations. Figure 3(a) shows the SEM image of a PD structure with similar dimensions as the previous test structure. In this PD structure, the D_offset_ is 200 nm. Figure 3b (i) shows the MFM image of the initial magnetization state when the transverse nanowire and the branch structure are magnetized in the +*y* and −*x* directions, respectively. As the magnetic field was increased along the +*x* direction, HH-ACW DW was injected and driven to the upper branch at a field strength of 65 Oe as shown in Fig. 3b (ii). This results in the change of the contrast of the upper branch from bright to dark. The final magnetization state for an array of PD structures as obtained from MFM imaging is shown in Fig. 3b (iii). Out of 16 structures in the array, 15 structures showed change in the magnetic contrast of the upper branch. Only one structure resulted in opposite trend. However, repeated measurements on the devices showed that the same device does not fail every time. The variation in the observed output may be due to thermal instability. Figure 3c (i) shows the MFM image of the initial state when the transverse nanowire and the branch structure are saturated in the −*y* and −*x* directions, respectively. As the magnetic field increased along the +*x* direction, HH-CW DW was injected and driven to the upper branch, as shown in Fig. 3c (ii). Similar observation was obtained for an array of such structures where barring one structure, the rest followed the same trend. The above MFM measurements were repeated on a single structure 20 times for both HH-ACW and HH-CW configurations and it was found that the DW always propagates along the upper branch in PD structure. The above experiments were carried out for the PU structure as well. The results indicate that the DW moves to the lower branch independent of its assigned chirality (supplementary Fig. S3). Thus by introducing an asymmetry in the device geometry, the DW can be deterministically propagated in a specific trajectory. Even though the final chirality of the DW at the bifurcation may be different from the initial orientation, setting an initial chirality allows us to compare results for two different initial conditions. Our results are independent of the initial or final chirality of the DW.

## Discussion

**Figure 4:**
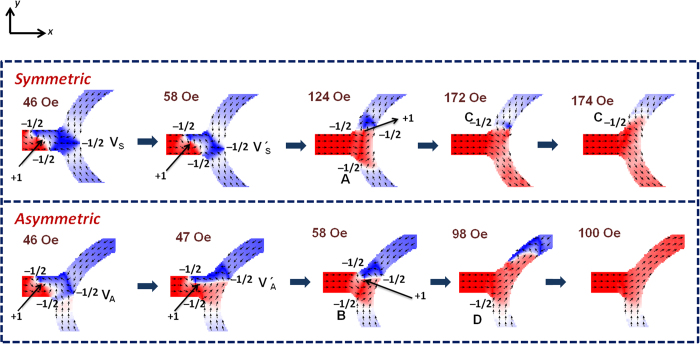
Domain wall evolution at the bifurcation. Simulations depicting DW evolution at the bifurcation for symmetric and asymmetric PD structure. An edge defect of topological charge −1/2 exists intrinsically at the bifurcation also referred to as vertex, the position of the topological charge at the vertex is slightly displaced in the lower branch for PD structure (V_A_) as compared to the symmetric structure (V_S_). As field is increased the edge defect at the vertex transforms to a vortex state. For the case of symmetric structure the vortex annihilates and the DW is nucleated in the opposite branch, the DW propagates in the branch which is opposite to topological edge defect of −1/2 (denoted as point C). For the PD structure, the VDW de-pins and moves along the upper branch, the path with less potential barrier. The DW moves in the branch opposite to topological edge defect of −1/2 (denoted as point D).

The DW dynamics were analyzed at the bifurcation of the branch structure to understand the underlying physics behind the deterministic DW motion. The dynamics are governed by the interaction of the injected DW with the stationary magnetic texture intrinsic to the branch structure at the bifurcation. The DWs can be characterized by a combination of various topological edge and bulk defects with particular winding numbers^20^. The arrangement of these edge defects defines the chirality of the DW. A VDW is composed of a bulk defect of a winding number +1 at the core and two edge defects of winding number −1/2. The total topological defects or charge of a magnetic system is always conserved^9^,^20^. Figure 4 shows the simulated magnetization states representing the evolution of an ACW VDWs at the bifurcation, for both the symmetric and asymmetric (PD) configurations. The nanowire length was kept below the DW fidelity length so as to potentially preserve the DW chirality. At the bifurcation, the magnetic texture with an edge defect of −1/2 exists in both the symmetric and asymmetric structures. However, the position of the −1/2 edge defect at the bifurcation is different for the two structures. For the symmetric structure, the position of the edge defect is at the centre (V_S_) with respect to the longitudinal nanowire whereas the edge defect is intrinsically pushed towards the lower branch (V_A_) for the asymmetric structure.

Consider the DW evolution at the bifurcation in the symmetric structure as magnetic field is increased. Upon the application of magnetic field, the VDW reaches the bifurcation. The interaction between injected VDW and the intrinsic magnetic texture at the bifurcation displaces the −1/2 defect towards the lower branch from Vs to V_s_′ when the field strength is 58 Oe. With further increase in the field strength to 124 Oe, the vortex core enters the upper branch and a rearrangement of topological charges take place. The VDW carries the −1/2 defect from point V_s_′ and leaves behind −1/2 defect at point A. When the magnetic field strength reaches 172 Oe, the VDW is annihilated leaving behind the −1/2 defect at point C. This process leads to depinning of the −1/2 defect at point A and propagation of a new DW in the lower branch. The DW nucleation in the lower branch requires higher field. The DW motion through the branch structure displaces the position of −1/2 defect from V_s_ to C.

Now consider the DW evolution at the bifurcation for the asymmetric structure as magnetic field is increased. Due to the geometrical asymmetry, the DW experiences a lower energy barrier towards the upper branch. As the DW approaches the bifurcation at field strength 47 Oe, the interaction with the magnetic texture at the vertex leads to the displacement of the edge defect towards the upper branch (V_a_′). With further increase in the field strength to 58 Oe, the vortex core enters the upper branch and a rearrangement of topological charges take place. The VDW carries the −1/2 defect from point V_a_′ and leaves behind −1/2 defect at point B. As the field is increased to 98 Oe, the VDW depins and propagates in the upper branch leaving behind −1/2 charge at point D. Thus the DW motion through the branch structure displaces the position of the −1/2 defect from V_A_ to D.

Comparison between the symmetric and asymmetric structures reveals that in the sequence the VDW propagates along the upper branch initially for both the structures as shown in the third image. However, the depinning field required is much larger for the case of symmetric structure than the asymmetric structure. It is because of the placement of the stationary −1/2 defect at the bifurcation after DW approaches it. In symmetric structure, −1/2 defect is displaced towards the lower branch, whereas the asymmetry displaces the −1/2 defect towards the upper branch in asymmetric structure. The vortex DW carries the −1/2 defect from the lower branch in case of symmetric structure and from the upper branch in the asymmetric structure, hence lesser field is required. The difference arises in the next sequence, where the VDW is annihilated for the case of symmetric structure and a new DW is nucleated which propagates in the lower branch. For the case of asymmetric structure the VDW itself depins and propagates in the upper branch. The asymmetry of the structure changes the potential landscape and plays a crucial role in guiding the DW to the respective branch. It is worth noting that the total topological defect or charge in the two structures before and after the rearrangement is conserved and remains as −1/2 in both symmetric and asymmetric structures. Also, the DW moves in the branch opposite to the −1/2 defect left by the DW transformation. The experimentally obtained MFM images and the simulated MFM images for the DW evolution at the bifurcation are shown in Supplementary Fig. S4.

We have carried out the above simulations for a CW VDW propagating along the symmetric and asymmetric PD structures (Supplementary Fig. S5). It is observed that the DW propagates along the upper branch. The mechanism is similar to the motion of ACW DW and involves the vortex carrying the −1/2 charge at the vertex, and leaving behind a −1/2 charge at the opposite corner of the bifurcation. We also noticed that the DW trajectory is independent of the polarity of the initial vortex core (Supplementary Fig. S6). Similar dynamics behavior at the bifurcation occurs for the PU structure.

**Figure 5:**
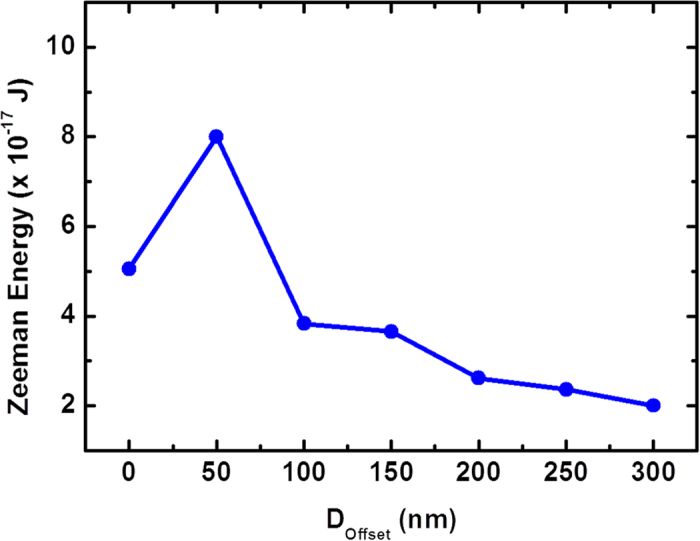
Potential energy barrier at the bifurcation. Plot of Zeeman energy needed to move the DW in one of the two branches as a function of D_offset_. There is a competition between two potential energy barriers one imposed due to selective trajectory and the other imposed due to geometrical asymmetry. Above 100 nm the potential barrier due to geometrical asymmetry in the PD structure is lower for the DW to move in the upper branch.

To quantify the effect of D_offset_, micromagnetic simulations for different values of D_offset_ were performed. The Zeeman (external field) energy, which is a measure of the pinning strength against the propagation of DW into one of the branches, was determined. Shown in Fig. 5 is the plot of Zeeman energy as a function of D_offset_ when a HH DW with ACW chirality is driven in the PD structure. For D_offset_ = 0, the structure is symmetrical, the DW moves towards the branch structure and gets pinned at the bifurcation. The DW experiences two potential barriers towards upper branch and lower branch, respectively. When the magnetic field is increased, the DW chooses to move along the lower branch according to the selective switching behavior. As D_offset_ is increased in the downward direction, the relative height of the two potential barriers varies *i.e.* the barrier along the upper branch is reduced and the barrier along the lower branch is increased. However, the DW still follows the selective switching behavior if it can overcome barrier along the lower branch. From our simulations we noted that for D_offset_ < 100 nm, the DW follows the same path as in the symmetrical structure (zero offset) *i.e.* it chooses to propagate along the lower branch. However above 100 nm, the DW cannot overcome the barrier towards the lower branch but sees a weak barrier along the upper branch and hence traverses through the upper branch. From the plot in Fig. 5 we observe that the Zeeman energy increases when D_offset_ = 50 nm, after which it reduces. This can be explained from the fact that below 100 nm, D_offset_ acts as a barrier and enhances the pinning against the propagation for the DW to move in to the lower branch and hence the Zeeman energy required is higher. When D_offset_ is increased above 100 nm, the barrier is higher towards the lower branch direction and lower towards the upper branch direction. Hence, the energy for DW propagation into the upper branch reduces. This effectively quantifies the effect of D_offset_.

In conclusion, we have demonstrated an approach to overcome the issue of stochastic DW trajectory in magnetic branch structures. Recent studies^10^,^11^,^21^ report that control of DW trajectory in artificial spin-ice lattice can potentially lead to monopole defect control. The selective trajectory of the DW in such network structures depends on the conservation of topological information between the vertices. Recently it was shown that the field required to switch such structures is large enough to suppress the transfer of topological information leading to lesser probability of path selectivity^22^. Thus in our structures a geometrical asymmetry was introduced in the branch structure which provides directional guidance to the DW propagation, leading to a deterministic path irrespective of the DW chirality. MFM scanning provides direct observation of the DW trajectory. The geometrically-guided DW trajectory can be extended to more complex structures, such as artificial spin ice lattice, where the hopping of magnetic charge can potentially be deterministically controlled. The deterministic trajectory of DW in magnetic network structures can be utilized in realizing analogous spintronics based applications such as non-volatile parallel processing and DW based logic devices^17^.

## Methods

### Device Fabrication

The nanostructures were fabricated on Si/SiO_2_ (300 nm) substrates. The substrates were cleaned with acetone and rinsed with iso-propyl alcohol for 20 mins each under ultrasonic agitation, and then blow dried with N_2_. A thin film stack of Ta/Ni_81_Fe_19_/Ta was deposited using ultra high vacuum magnetron sputtering techniques. The wafers were then spin-coated with negative resist and the nanostructured patterns were written by using electron beam lithography. The pattern transfers were completed using Ar ion milling technique. The negative resist was subsequently removed by using oxygen plasma stripper.

### Micromagnetic Simulations

The Object Oriented Micromagnetic Framework (OOMMF) program developed by the National Institute of Standards and Technology^19^ was used to perform the micromagnetic simulations. The chosen material parameters for our magnetic thin film Ni_81_Fe_19_ are, saturation magnetization, *M*_*S*_ = 8.6 × 10^5 ^Am^−1^, anisotropy constant, *K* = 0, exchange stiffness constant *A* = 1.3 × 10^−11 ^Jm^−1^, and Gilbert damping constant, *α* = 0.01. A mesh size of 5 × 5 × 5 nm^3^ was employed for all simulations.

### Magnetic Force Microscopy

MFM scanning was carried out by using DI Dimension 5000 system with commercial low moment magnetic tips. A lift-scan height of 100 nm was used during the measurements to obtain an optimised separation of magnetic-topography signal. MFM images were captured after applying and removing external magnetic field, hence the magnetic nanostructure samples were considered as quasi-remnant state. The magnetic images were captured in phase detection mode. Bright contrast in the scanned MFM image is defined as repulsive interaction and net negative magnetic charge.

## Additional Information

**How to cite this article**: Sethi, P. *et al.* Direct observation of deterministic domain wall trajectory in magnetic network structures. *Sci. Rep.* **6**, 19027; doi: 10.1038/srep19027 (2016).

## Supplementary Material

Supplementary Information
