# Supplementary material for: Direct observation of deterministic domain wall trajectory in magnetic network structures
Source: Sci Rep. 2016 Jan 12;6:19027. doi: 10.1038/srep19027 (PMC4709518; doi:10.1038/srep19027)
Supplement: Supplementary Information [file srep19027-s1.pdf]

Supplementary Information

Direct observation of deterministic domain wall trajectory in magnetic network structures

P. Sethi[1, 2], C. Murapaka[1], S. Goolaup[1], Y. J. Chen[2], S. H. Leong[2] and W. S. Lew[1]*

[1]School of Physical & Mathematical Sciences, Nanyang Technological University
21 Nanyang Link, Singapore 637371

*[2]Data Storage Institute, (A*STAR) Agency for Science, Technology and Research,*
DSI Building, 5 Engineering Drive 1, Singapore 117608

*Corresponding author: wensiang@ntu.edu.sg

Figure S1 Simulations depicting the injection of vortex domain wall (VDW) with a specific chirality according to the transverse nanowire magnetization

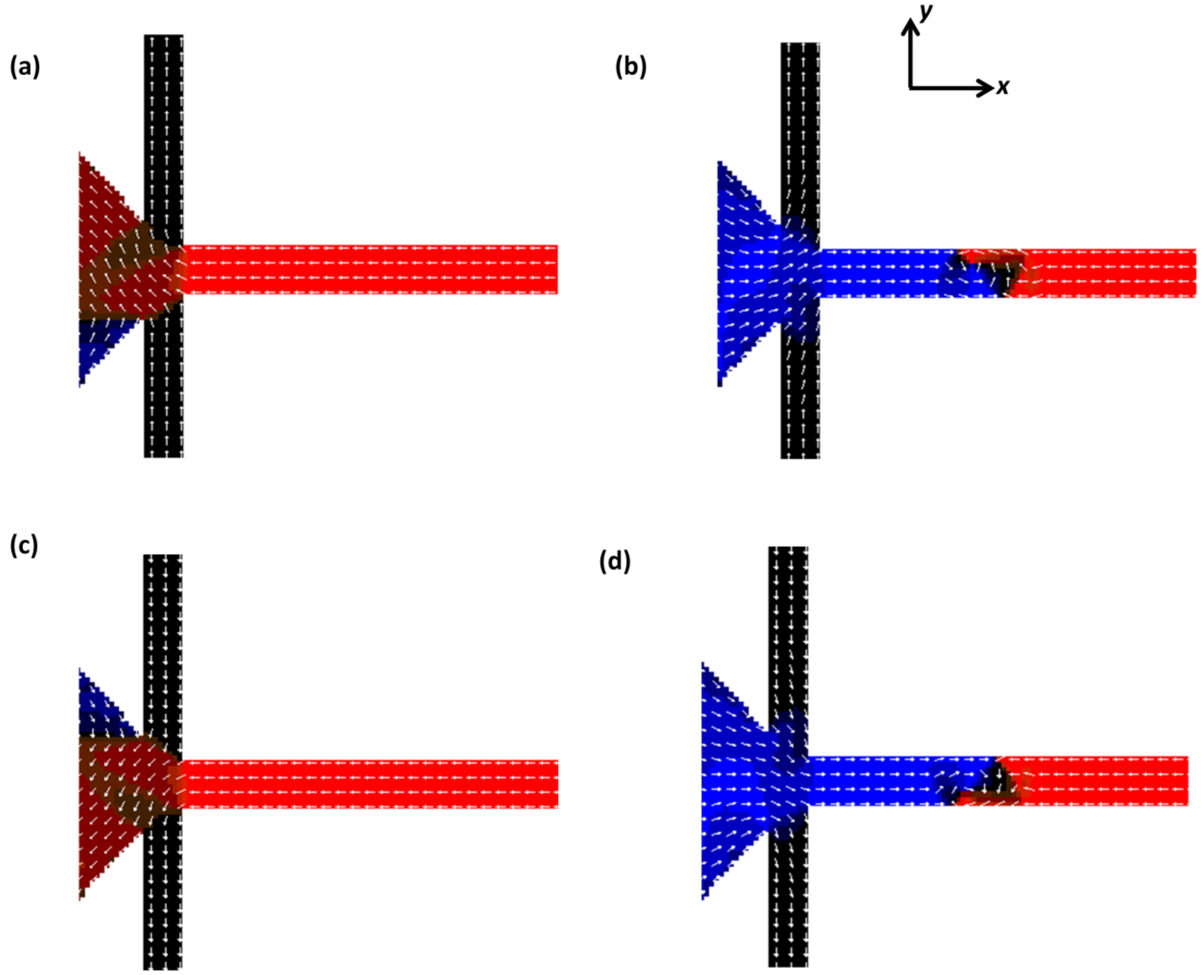

(a) Initial magnetization configuration when transverse nanowire and the longitudinal nanowires are magnetized along +*y* and –*x* directions, respectively. (b) Final magnetization configuration when the magnetic field is increased along +*x* direction, injecting head-to-head (HH) domain wall (DW) with anti-clockwise (ACW) chirality (c) Initial magnetization configuration when transverse nanowire and the longitudinal nanowires are magnetized along *–y and –x* directions, respectively (d) Final magnetization configuration when magnetic field is increased along +*x* direction, injecting HH DW with clockwise chirality

Figure S2 Simulations showing motion of DW in asymmetric branch structure.

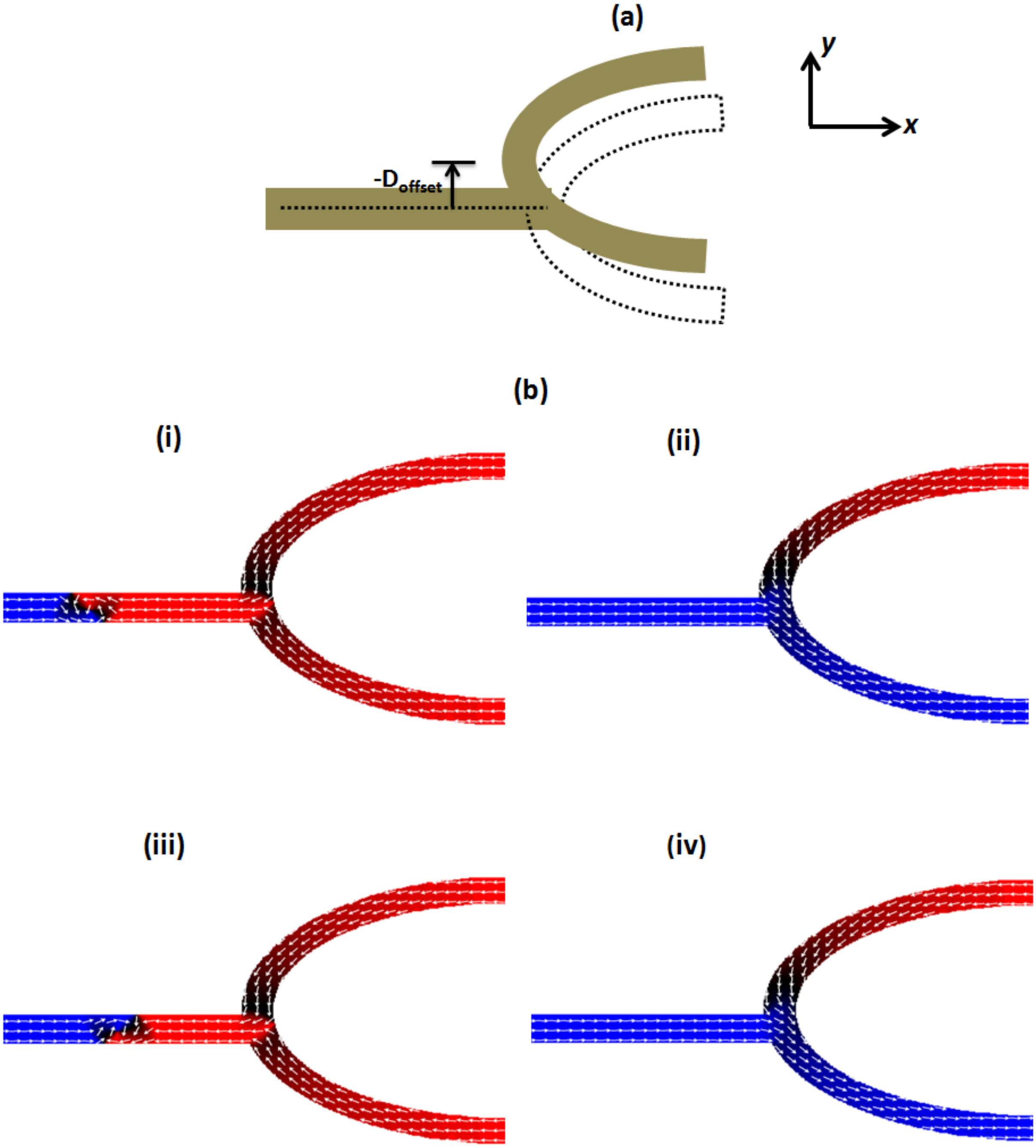

(a) Schematic of ‘pull-up’ (PU) structure, where output branch is displaced in the +*y* direction and the offset is labeled –D_{offset}. OOMMF simulations (b-i) Initial magnetization configuration of the PU structure when HH-ACW is injected in the

nanowire. (b-ii) Final magnetization configuration of the PU structure depicting DW moving to the lower branch. (b-iii) Initial magnetization configuration of the PU structure when HH-CW is injected in the nanowire. (b-iv) Final magnetization configuration of the PU structure depicting DW moving to the lower branch.

Figure S3 Direct observation of vortex DW trajectory in PU structure.

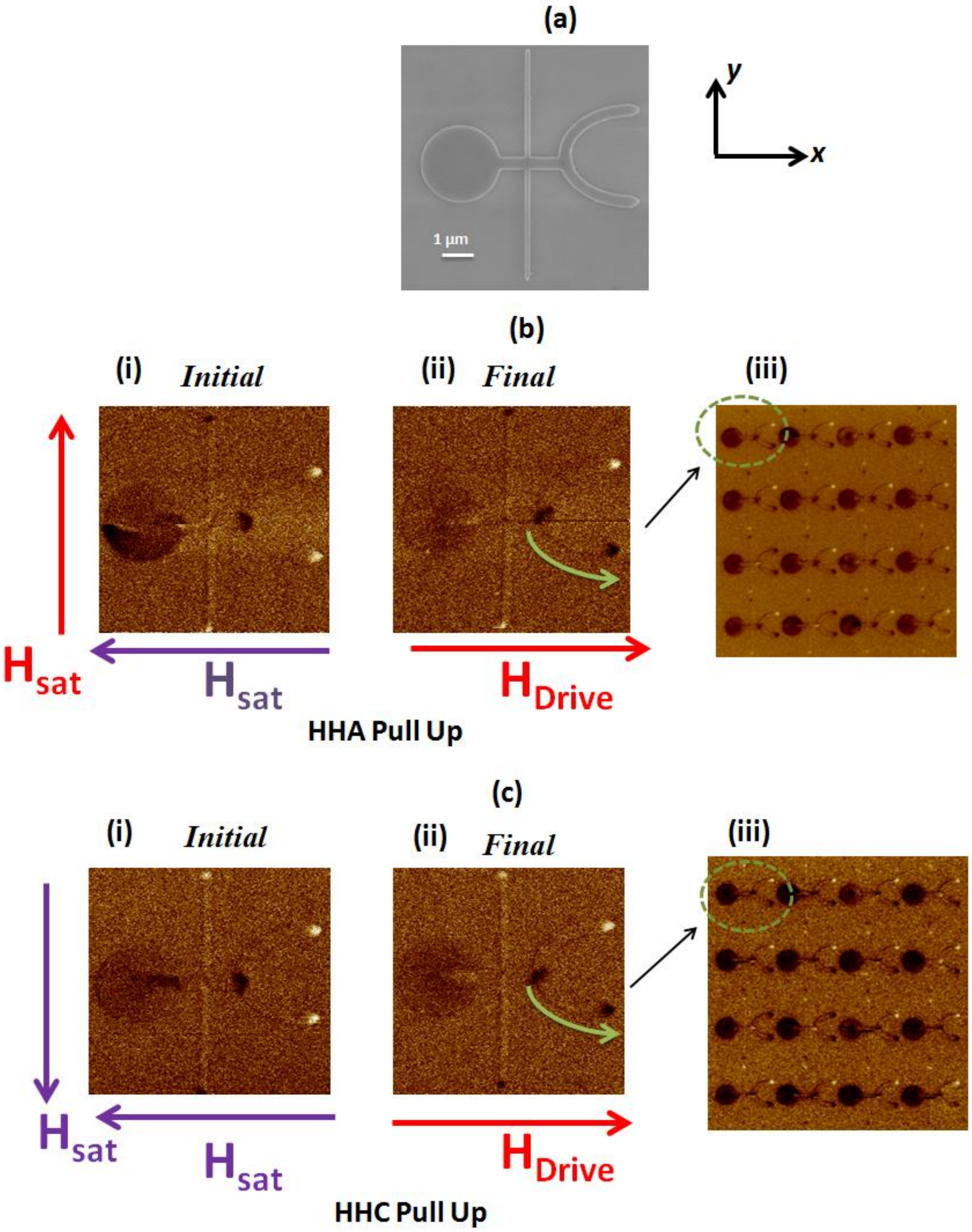

(a) SEM image depicting the PU structure, the output branch is displaced in the +*y* Direction by 200 nm. MFM imaging of the PU structure. (b-i) Initial magnetization configuration when transverse nanowire is saturated in the +*y*-direction and the output branch is saturated in the –*x* direction. (b-ii) Final magnetization configuration when HH-ACW is driven through the branch structure. (b-iii) Final MFM image of an array

of PD structures, here all devices follow the above trend indicating the DW motion along the lower branch. (c-i) Initial magnetization configuration when transverse nanowire is saturated in the -*y*-direction and the output branch is saturated in the –*x* direction. (c-ii) Final magnetization configuration when HH-CW is driven through the branch structure. (c-iii) Final MFM image of an array of PU structures, here all devices follow the above trend of motion along the lower branch.

Figure S4 Magnetic force microscopy imaging at the bifurcation and the corresponding simulated configurations

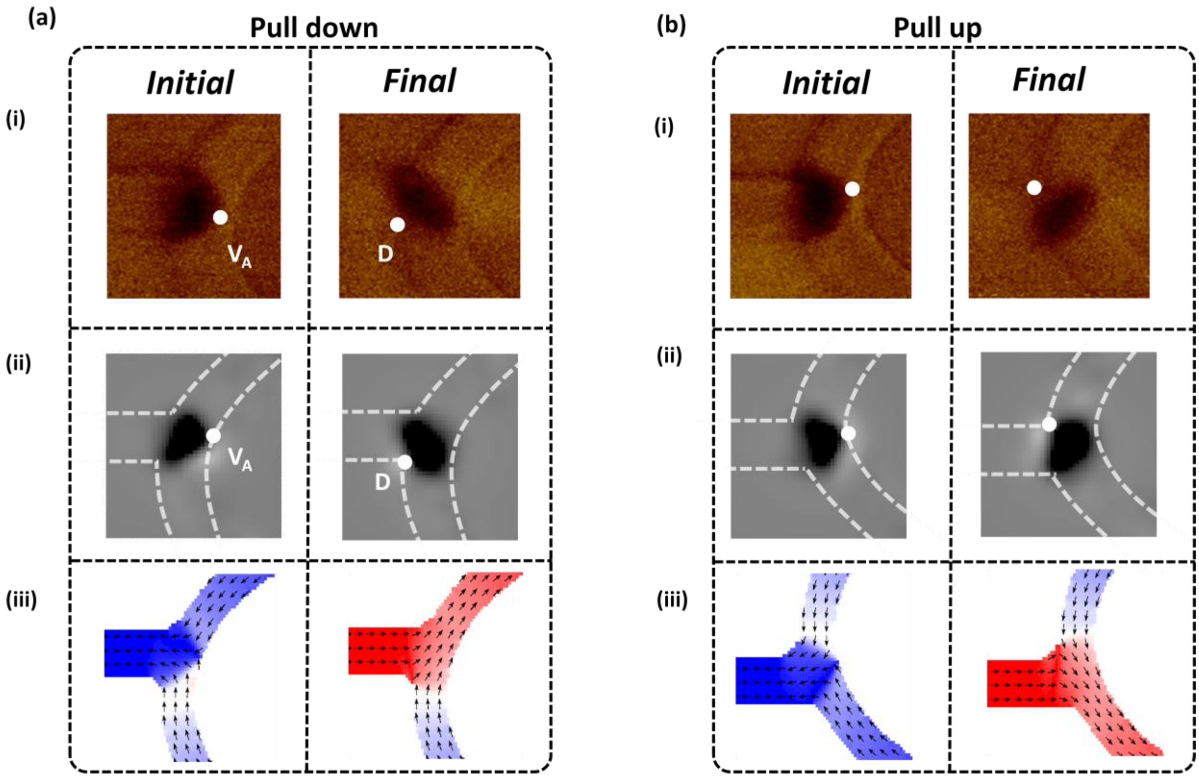

Domain wall evolution at the bifurcation (b-i) MFM image of the initial and final magnetization state at the bifurcation for the PD structure as a DW is driven through it. Topological charge at the Vertex (V_A) is intrinsically displaced towards the lower branch. Point D represents the edge defect left by the DW transformation. (b-ii) Simulated MFM contrast of the initial and final magnetization states at the bifurcation for the PD structure (b-iii) Simulated spin configuration of the initial and final magnetization states at the bifurcation for the PD structure (c-i) MFM image of the initial and final magnetization state at the bifurcation for the PU structure as a DW is driven through it. The topological charge at the vertex is intrinsically displaced towards the upper branch. (c-ii) Simulated MFM contrast of the initial and final magnetization states at the bifurcation for the PU structure as a DW is

driven through it. (c-iii) Simulated spin configuration of the initial and final magnetization states at the bifurcation for the PU structure

Figure S5 Evolution of clockwise vortex DW at the bifurcation

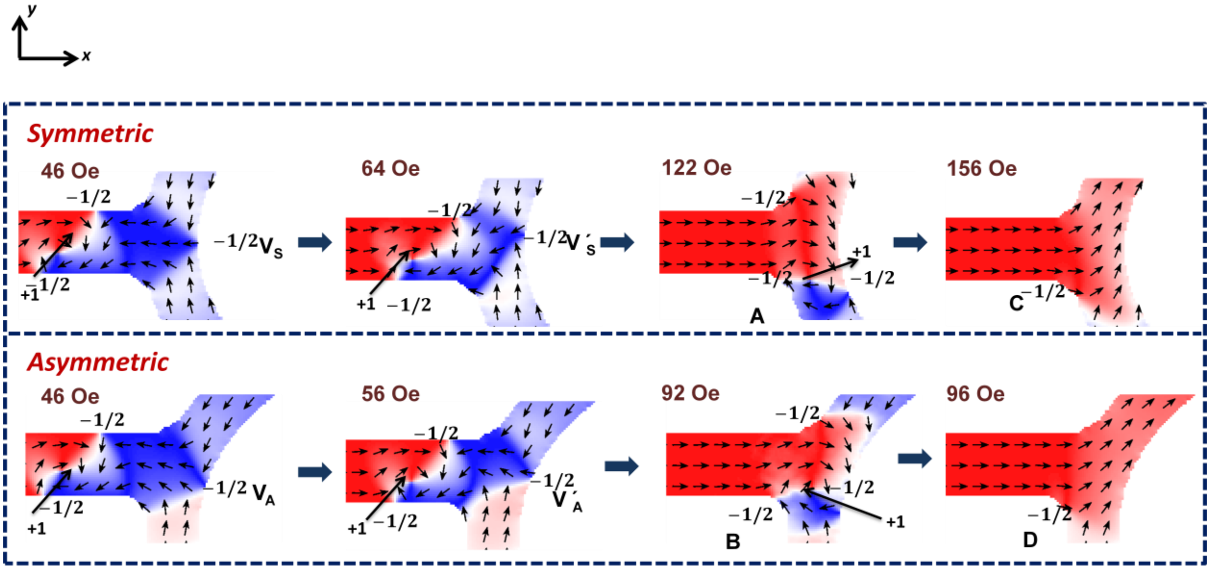

Simulations depicting DW evolution at the bifurcation for symmetric and asymmetric PD structure. An edge defect of topological charge $-1/2$ exists intrinsically at the bifurcation labeled as vertex, the position of the vertex is slightly displaced in the lower branch for PD structure (V_A) as compared to the symmetric structure (V_S). As field is increased the edge defect at the vertex transforms to a vortex state. For the case of symmetric structure the vortex annihilates and the DW is nucleated in the opposite branch, the DW propagates in the branch which is opposite to topological edge defect of $-1/2$ (denoted as point C). For the PD structure, the VDW de-pins and moves along the upper branch, the path with less potential barrier. The DW moves in the branch opposite to topological edge defect of $-1/2$ (denoted as point D)

Figure S6 Simulations depicting motion vortex DW with positive and negative core polarities

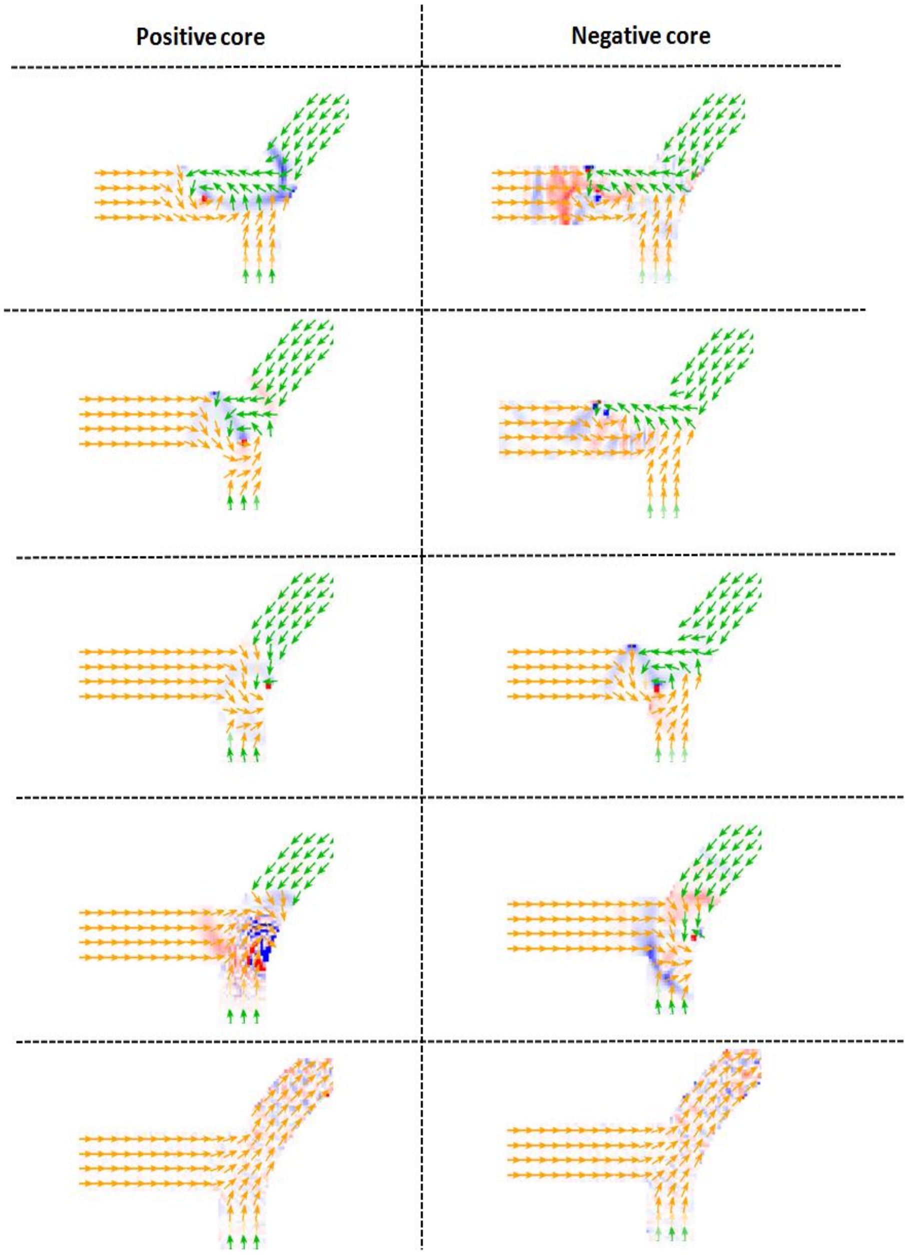

Simulations depicting motion of vortex DW with positive and negative core polarities. The DW moves in the upper branch irrespective of the initial core polarity. For an ACW DW, the positive polarity is maintained throughout its motion. However, the negative core polarity transforms to a positive polarity near the bifurcation.
